# Bromocriptine treatment in patients with peripartum cardiomyopathy and right ventricular dysfunction

**DOI:** 10.1007/s00392-018-1355-7

**Published:** 2018-08-18

**Authors:** Arash Haghikia, Johannes Schwab, Jens Vogel-Claussen, Dominik Berliner, Tobias Pfeffer, Tobias König, Carolin Zwadlo, Valeska Abou Moulig, Annegret Franke, Marziel Schwarzkopf, Philipp Ehlermann, Roman Pfister, Guido Michels, Ralf Westenfeld, Verena Stangl, Uwe Kühl, Edith Podewski, Ingrid Kindermann, Michael Böhm, Karen Sliwa, Denise Hilfiker-Kleiner, Johann Bauersachs

**Affiliations:** 1 Department of Cardiology and Angiology, Hannover Medical School, Carl-Neuberg-Str. 1, 30625 Hannover, Germany; 2 Department of Cardiology, Charité-Universitätsmedizin Berlin, Campus Benjamin Franklin, Hindenburgdamm 30, 12203 Berlin, Germany; 3 Universitätsklinik für Innere Medizin 8-Schwerpunkt Kardiologie und Institut für Radiologie und Nuklearmedizin, Klinikum Nürnberg Süd, Paracelsus Medizinische Privatuniversität Nürnberg, Breslauer Str. 201, 90471 Nuremberg, Germany; 4 Institute for Diagnostic and Interventional Radiology, Hannover Medical School, Carl-Neuberg-Str. 1, 30625 Hannover, Germany; 5 Faculty of Medicine, University Leipzig, Clinical Trial Centre (KKS), ZKS Leipzig, Haertelstr. 16-18, 04103 Leipzig, Germany; 6 Department of Cardiology, Angiology, and Pneumology, University of Heidelberg, Im Neuenheimer Feld 410, 69120 Heidelberg, Germany; 7 Department of Cardiology, Pulmonology, and Vascular Medicine, University of Cologne, Kerpenerstr. 62, 50937 Cologne, Germany; 8 Department of Cardiology, Pulmonology, and Vascular Medicine, Medical Faculty, University Duesseldorf, Duesseldorf, Germany; 9 Department for Cardiology and Angiology, Center for Cardiovascular Research (CCR), Charité-Universitätsmedizin Berlin, Charitéplatz 1, 10117 Berlin, Germany; 10 Department of Internal Medicine III, University Hospital of the Saarland, 66421 Homburg, Saar Germany; 11 Faculty of Health Sciences, Hatter Institute of Cardiology Research in Africa, 2 Anzio Road, Chris Barnard Building, 4th Floor, Observatory, Cape Town, 7925 South Africa

**Keywords:** Peripartum cardiomyopathy, Right ventricular dysfunction, Bromocriptine therapy

## Abstract

**Background:**

Right ventricular (RV) dysfunction predicts adverse outcome in peripartum cardiomyopathy (PPCM). We recently demonstrated beneficial effects associated with the prolactin release inhibitor bromocriptine at different doses when added to standard heart failure therapy in PPCM. Here, we evaluated for the first time the therapeutic potential of bromocriptine particularly in PPCM patients with RV involvement.

**Methods:**

In this study, 40 patients with PPCM were included, of whom 24 patients had reduced RV ejection fraction (RVEF < 45%). We examined the effect of short-term (1W: bromocriptine, 2.5 mg, 7 days, *n* = 10) compared with long-term bromocriptine treatment (8W: 5 mg for 2 weeks followed by 2.5 mg for another 6 weeks, *n* = 14) in addition to guideline-based heart failure therapy in patients with an initial RVEF < 45% on the following outcomes: (1) change from baseline (Δ delta) in RVEF, (2) change from baseline in left ventricular EF (LVEF), and (3) rate of patients with full LV recovery (LVEF ≥ 50%) and (4) rate of patients with full RV recovery (RVEF ≥ 55%) at 6-month follow-up as assessed by cardiac magnetic resonance imaging.

**Results:**

Reduced RVEF at initial presentation was associated with a lower rate of full cardiac recovery at 6-month follow-up (patients with RV dysfunction: 58% vs. patients with normal RV function: 81%; *p* = 0.027). RVEF increased from 38 ± 7 to 53 ± 11% with a delta-RVEF of + 15 ± 12% in the 1W group, and from 35 ± 9 to 58 ± 7% with a Δ RVEF of + 23 ± 10% in the 8W group (Δ RVEF 1W vs 8W: *p* = 0.118). LVEF increased from 25 ± 8 to 46 ± 12% with a Δ LVEF of + 21 ± 11% in the 1W group, and from 22 ± 6 to 49 ± 10% with a Δ LVEF of + 27 ± 9% in the 8W group (Δ LVEF 1W vs 8W: *p* = 0.211). Full LV recovery was present in 50% of the 1W group and in 64% of the 8W group (*p* = 0.678). Full RV recovery was observed in 40% of the 1W group and in 79% of the 8W group (*p* = 0.092).

**Conclusions:**

Despite overall worse outcome in patients with RV dysfunction at baseline, bromocriptine treatment in PPCM patients with RV involvement was associated with a high rate of full RV and LV recovery, although no significant differences were observed between the short-term and long-term bromocriptine treatment regime. These findings suggest that bromocriptine in addition to standard heart failure therapy may be also effective in PPCM patients with biventricular impairment.

**Electronic supplementary material:**

The online version of this article (10.1007/s00392-018-1355-7) contains supplementary material, which is available to authorized users.

## Introduction

Peripartum cardiomyopathy (PPCM) is a major cause of pregnancy-related heart failure with a considerable rate of morbidity and mortality with a considerable risk of life-threatening arrhythmia [1–3]. Myocardial microvascular damage caused by a cleaved fragment of the nursing hormone prolactin is considered an essential pathomechanism of PPCM [4]. Among determinants of poor outcome, very low baseline left ventricular (LV) ejection fraction (LVEF < 25%) and LV dilatation were identified in studies from different regions [5–7]. Moreover, we and others recently found that about one-third of the patients also manifest right ventricular (RV) dysfunction at initial presentation which is a predictor of adverse outcome [8, 9]. Importantly, most of our patients with impaired RV function also displayed lower LV ejection fraction and more pronounced LV enlargement indicative of an overall more severe cardiac pathology. Thus, RV involvement may reflect a specific subtype of PPCM with a more severe course of disease and may, therefore, require distinct and more intensive treatment strategies. However, to date the effect of currently available treatment options on RV function in PPCM has not been evaluated.

We recently demonstrated in a multicenter, randomized controlled trial that treatment with the prolactin release inhibitor bromocriptine in addition to guideline-recommended heart failure therapy is safe and improves LV function and clinical outcome in PPCM [10]. Our results suggested that a short and low-dose bromocriptine therapy aiming to suppress prolactin release is sufficient in most cases. However, we also observed a trend for higher rate of full cardiac recovery in patients with a more severe initial condition when treated longer with a higher dosage of bromocriptine suggesting additional protective properties of bromocriptine beyond its prolactin suppressing effects in distinct PPCM subgroups.

In the present substudy, we examined the effects of prolonged bromocriptine treatment compared to short-term treatment in addition to guideline-recommended heart failure therapy [11] on RV and LV function, and the rate of full cardiac recovery in PPCM patients with biventricular dysfunction at initial presentation.

This is the first study to evaluate the impact of a therapeutic strategy on RV function in PPCM. The results may help to improve treatment options for PPCM patients with biventricular dysfunction and more severe clinical course.

## Materials and methods

### Study design

This is a substudy of a recently reported prospective, randomized, controlled trial, conducted in 12 participating centers in Germany [10]. The trial (ClinicalTrials.gov. NCT00998556) was approved by the ethics committees at each participating institution and all patients gave written informed consent. The detailed study design has been described previously [12].

### Study population

From June 2010 until September 2015, 140 patients at 12 centers were screened for eligibility and 68 patients were enrolled in the trial. In brief, eligibility requirements at screening included an age of at least 18 years, new onset PPCM within a time-window of 5-month postpartum according to the definition by the Study Group on PPCM from the Heart Failure Association of the European Society of Cardiology (ESC) [1] and a substantial LV dysfunction with a LVEF ≤ 35% as assessed by echocardiography. Exclusion criteria included a preexisting cardiac disease or previous cardiac surgery or percutaneous coronary intervention, any preexisting serious conditions, history of alcohol and/or any other drug abuse and contraindication to the planned therapy.

**Fig. 1:**
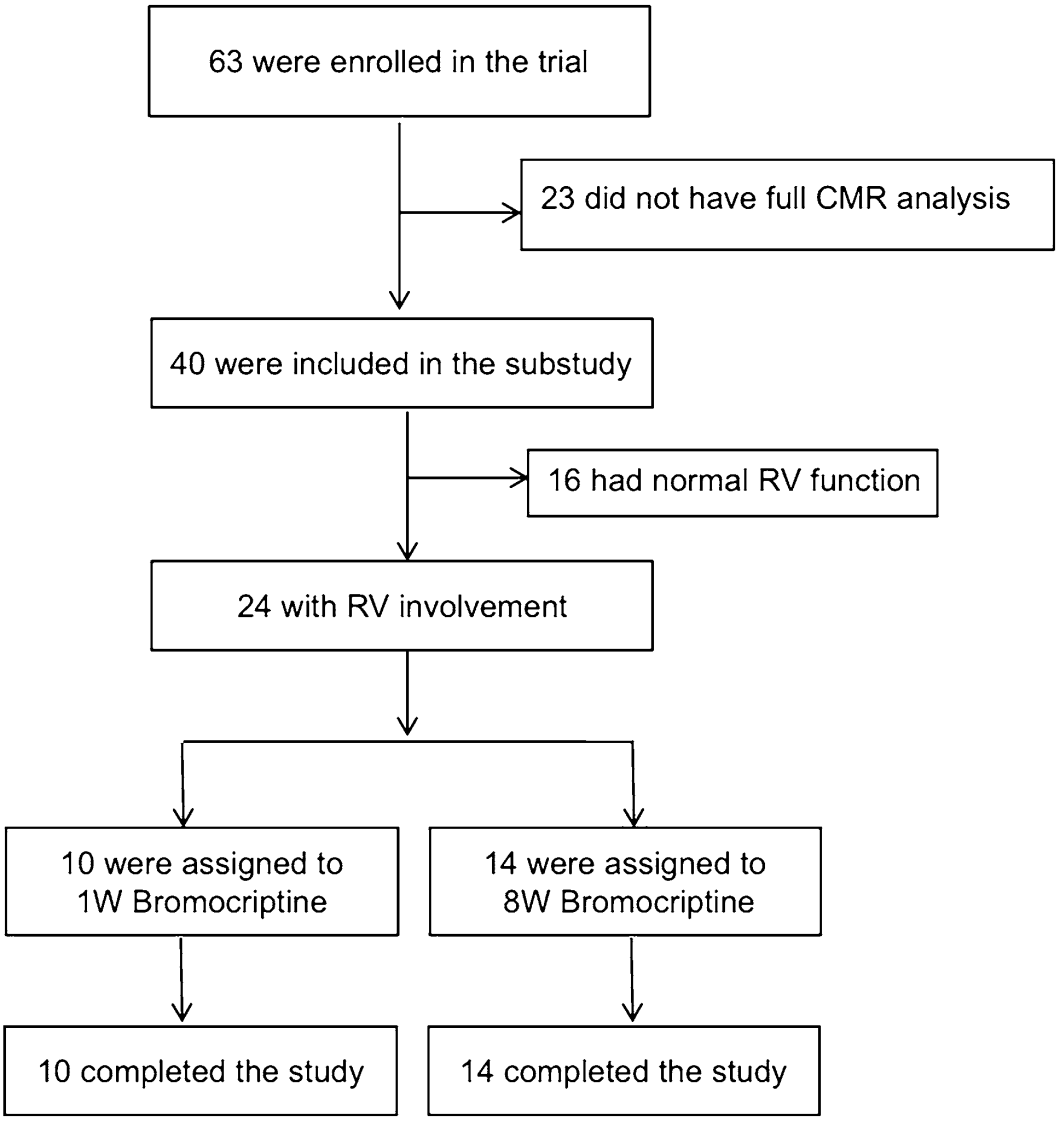
Flowchart illustrates the study profile. Initially, 68 patients fulfilled the trial inclusion criteria with 40 patients with full CMR assessments of the right ventricle at baseline and at 6-month follow-up. Of these patients 24 had reduced RV function with a RVEF < 45%, of whom 10 patients were randomly assigned to receive 2.5 mg bromocriptine for 1 week (1W group) and 14 patients to receive 5 mg bromocriptine for 2 weeks followed by 2.5 mg for another 6 weeks (8W group) in addition to guideline-based heart failure medication

In this substudy, we included only patients with cardiac magnetic resonance imaging (CMR) assessments of the right ventricle at baseline (recruited in eight centers) and at 6-month follow-up (*n* = 40). Of these patients 24 had reduced RV function with a RVEF < 45%, of whom ten patients were randomly assigned to receive 2.5 mg bromocriptine for 1 week (1W group) and 14 patients to receive 5 mg bromocriptine for 2 weeks followed by 2.5 mg for another 6 weeks (8W group) in addition to guideline-based heart failure medication (study profile is shown in Fig. 1). Detailed baseline characteristics of all 40 patients are provided in Suppl. Table 1.

### Trial outcome

For the purpose of the present study, we performed a post hoc analysis of patients with an initial RVEF < 45% on the following outcomes: (1) change of global RVEF, (2) change of global LVEF, and (3) rate of patients with full RV recovery (RVEF ≥ 55%) and (4) rate of patients with full LV recovery (LVEF ≥ 50%) after 6-month follow-up as examined by CMR.

### Image analysis

Image analysis was performed by a core lab run by an experienced radiologist and cardiologist together in consensus using dedicated CMR evaluation software (cmr42, Circle Cardiovascular Imaging, Calgary, Alberta, Canada). The investigators were blinded to the patients` therapy. Standard methods were used to calculate LV and RV end-diastolic and end-systolic volume, the resulting stroke volume and the ejection fraction from the cine SSFP images in the short-axis views images by manually tracing ventricular endocardial and epicardial contours in end-diastole and end-systole as described previously [8].

### Statistical analysis

Baseline characteristics are presented as means ± SD for continuous variables and percentages for categorical variables. Continuous data were expressed as mean ± SD or median and range. Comparison of means and proportions between two groups was performed by independent *t* test and Fisher exact test, respectively. Wilcoxon rank-sum test was used if data were not normally distributed.

Absolute changes from baseline to follow-up in LVEF and RVEF were compared between treatment groups using analysis of covariance with follow-up EF as dependent variable, the treatment arm as fixed factor, and baseline EF as covariate as previously described [10].

Final cardiac status of patients after 6-month follow-up was evaluated in patients with initial RVEF < 45%: ‘full RV recovery’ was predefined as last RVEF ≥ 55%, ‘full LV recovery’ as last LVEF ≥ 50%, ‘partial RV recovery’ as last RVEF ≥ 45% and < 55%, ‘partial LV recovery’ as last LVEF ≥ 35% and < 50%, ‘no RV recovery’ as last RVEF < 45% and ‘no LV recovery’ as last LVEF < 35%. Analyses were performed using IBM SPSS Statistic version 25 (IBM Corp., Armonk, NY, USA). All *p* values are two sided, and a *p* value of < 0.05 was considered significant.

## Results

### Baseline characteristics and standard heart failure treatment

Diagnosis was made 1.4 ± 2 months after delivery and average time between first symptoms and diagnosis was 3 weeks with no difference between groups. All patients were randomized postpartum. Randomization was done no more than 7 days after first diagnosis of PPCM.

The characteristics of all randomized patients at baseline are provided in Suppl. Table S1. Only renal function differed significantly between patients with RV dysfunction and normal RV function with lower renal function in patients with RV dysfunction (serum creatinine in patients with RV dysfunction: 0.90 ± 0.1 vs normal RV function: 0.76 ± 0.1, *p* < 0.015). Otherwise, the patients were balanced with respect to most clinical and epidemiological characteristics such as age, gravida, parity, race, body mass index, hemodynamics, cardiac function, New York Heart Association class, cardiovascular and pregnancy-related risk factors. Importantly, use of guideline-based heart failure therapy including ACE-inhibitors or angiotensin receptor blocker (ARB), beta-blockade and mineralocorticoid receptor antagonist (MRA) [11] during the study was high and did not differ between both groups (Suppl. Table S1). A comparison of baseline characteristics in patients with reduced RV function between the two treatment groups is provided in Suppl. Table S2.

### Baseline CMR characteristics

Comparison of baseline CMR findings on the left and right ventricular function revealed that patients with RV dysfunction at initial presentation also had significantly lower LV function and more pronounced LV dilatation (Suppl. Table S3). Analyses of RV parameters indicated that beside lower RVEF significantly lower stroke volume and greater RV enlargement were also present in patients with biventricular PPCM (Suppl. Table S3). We further evaluated whether baseline CMR characteristics in patients with reduced RV function were balanced between the two treatment groups (1W vs 8W bromocriptine treatment). As illustrated in Suppl. Table S4, no significant differences were observed for any LV or RV parameter.

### Outcome of patients with biventricular compared with isolated left ventricular dysfunction

**Fig. 2:**
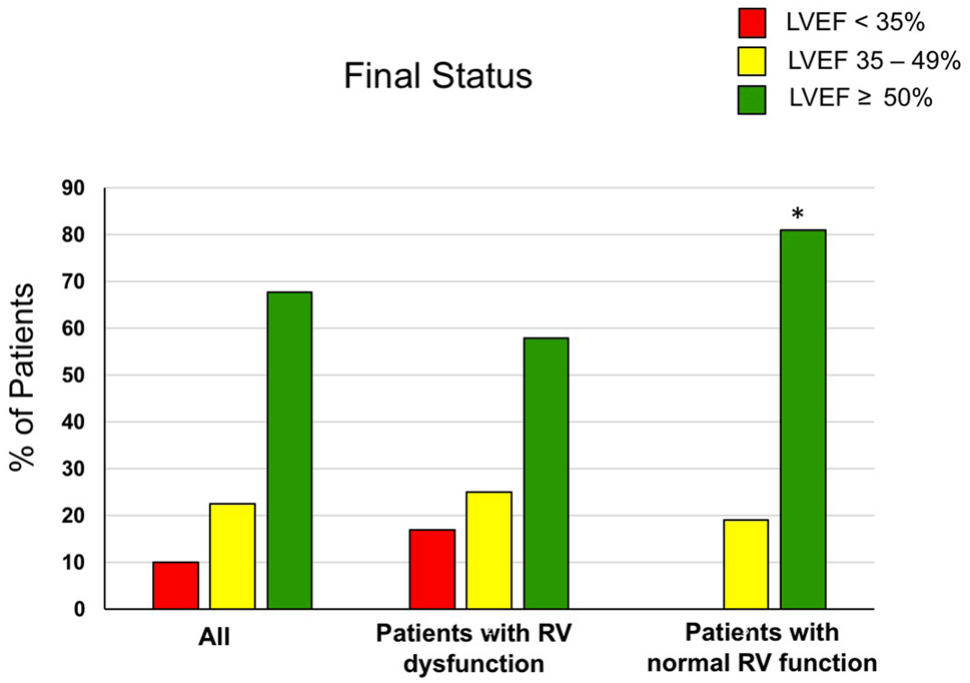
Outcome of all 40 patients at 6 months. Left ventricular ejection fraction according to predefined categories in all patients (left bars) and subdivided between patients with reduced RVEF (center bars) and normal RVEF (right bars) at baseline. Red bars illustrate the percentage of patients with no recovery (final LVEF < 35), yellow bars illustrate the percentage of patients with partial recovery (final LVEF 35% to < 50%) and green bars depict percentage of patients with full recovery (final LVEF ≥ 50%). Note that the rate of full recovery was higher among patients with normal RV function, *n* = 16, compared to patients with RV dysfunction, *n* = 24, at baseline (*indicates *p* < 0.05 compared to patients with RV dysfunction)

After 6-month follow-up, 68% of all study patients showed full functional LV recovery (LVEF ≥ 50%, green bars), 22% partial recovery (LVEF from 35% and < 50%, yellow bars) and 10% no recovery (LVEF < 35%, red bars) (Fig. 2). Among patients with RV dysfunction at baseline, the respective rates were 58% for full functional recovery, 25% for partial recovery, and 17% for no recovery. In comparison, patients without RV dysfunction at baseline showed a significantly higher rate of full LV recovery (81%) (two-sided Fisher exact test *p* = 0.027 with an OR of 0.093 (95% confidence interval 0.01054–0.8268) compared to patients with RV dysfunction) (Fig. 2). Notably, none of the patients with normal RV function at baseline had a LVEF < 35% at follow-up. The rate of patients for partial recovery was 19% (*p* = 0.717 compared to patients with RV dysfunction).

### Change in right ventricular function of all patients

**Fig. 3:**
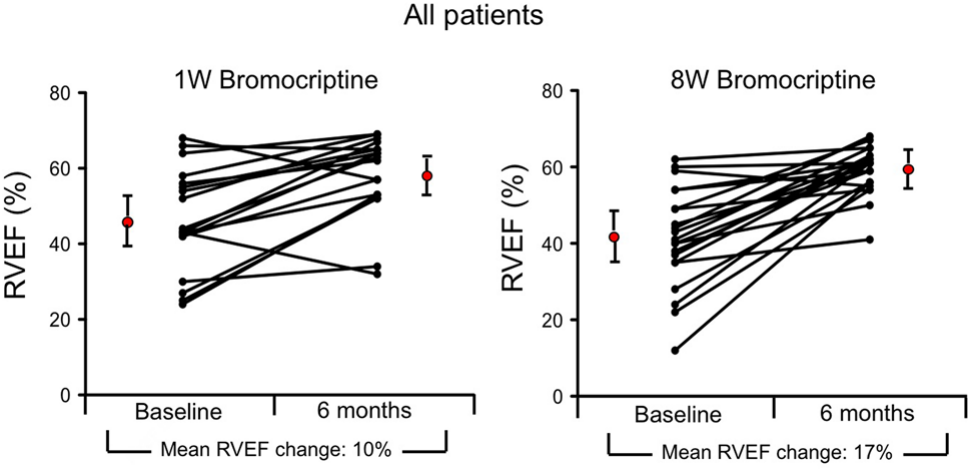
Change of global right ventricular ejection fraction (RVEF) from baseline to 6-month follow-up determined by CMR of all patients. Graph depicts individual courses of RVEF change from baseline to 6-month follow-up in the patients treated for 1 week with Bromocriptine (1W group, *n* = 18, left graph) and those treated for 8 weeks (8W group, *n* = 22, right graph) with a between-groups difference at 6-month follow-up of 7% in favor of the 8W group, *p* = 0.308

The mean RVEF increased from a mean of 48 ± 12% at baseline to 58 ± 10% at 6 months in the 1W group (*n* = 18), and from 42 ± 13 to 59 ± 6% in the 8W group (*n* = 22). Although delta RVEF was higher in the 8W group (Δ 17 ± 12%) compared with the 1W group (Δ 10 ± 11%) with a between-groups difference at 6-month follow-up of 7%, this was not statistically significant (*p* = 0.308). Individual courses are presented in Fig. 3.

### Change in right ventricular function of patients with biventricular dysfunction

**Fig. 4:**
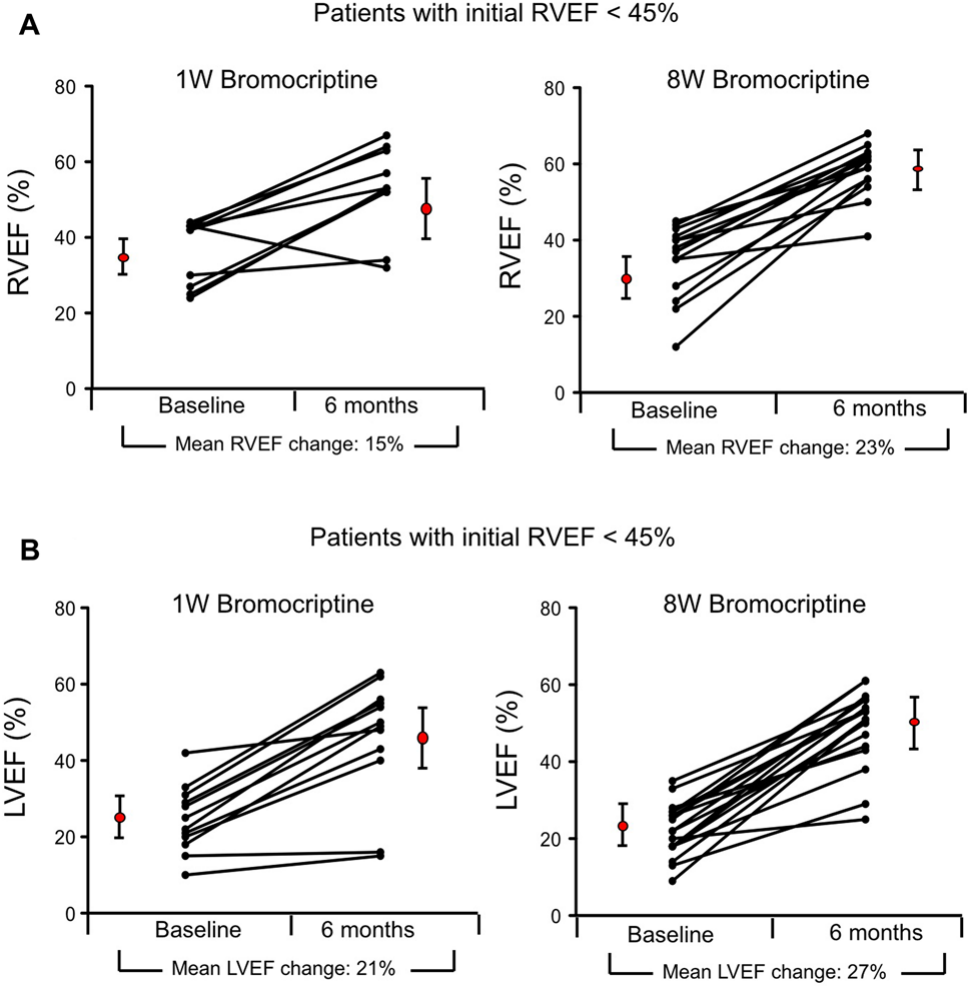
Individual courses of **a** RVEF and **b** LVEF change from baseline to 6-month follow-up for the subgroup of patients with RVEF < 45% at study entry with 1W bromocriptine (*n* = 10, left graphs) compared to 8W bromocriptine treatment (*n* = 14, right graphs). **a** Depicts individual courses of RVEF with a between-groups difference at 6-month follow-up of 8% in favor of the 8W group, *p* = 0.118. **b** Depicts individual courses of LVEF with a between-groups difference at 6-month follow-up of 6% in favor of the 8W group, *p* = 0.211

To evaluate effects of the two treatment concepts in more severely diseased patients with RV involvement, additional analysis was performed focusing on only patients in whom baseline RVEF was < 45%. The change of RVEF in these patients was compared between the two treatment groups. As shown in Fig. 4a, the RVEF increased from a mean of 38 ± 7% at baseline to 53 ± 11% at 6 months in the 1W group, and from 35 ± 9 to 58 ± 7% in the 8W group. The delta RVEF in the 8W group (Δ + 23 ± 10%) tended to be higher compared with delta RVEF of the 1W group (Δ + 15 ± 12%). However, the difference did not reach statistical significance (Δ RVEF 1W vs 8W: *p* = 0.118). Based on the observed effects, a minimum of 28 patients per group was estimated to achieve significant p with an 80% power.

### Change in left ventricular function of patients with biventricular dysfunction

In patients with baseline RVEF < 45%, the change of LVEF was compared between the two treatment groups. As shown in Fig. 4b, the LVEF increased from a mean of 25 ± 8% at baseline to 46 ± 12% at 6 months in the 1W group, and from 22 ± 6 to 49 ± 10% in the 8W group. Although a tendency towards higher delta LVEF was observed in the 8W group (8W group: Δ + 27 ± 9% vs 1W group: Δ + 21 ± 11%), the difference did not reach statistical significance (8W vs 1W: *p* = 0.211). Based on the observed effects, a minimum of 42 patients per group would result in an estimated significant p with an 80% power.

### Final right and left ventricular status of patients with biventricular dysfunction with regard to bromocriptine treatment duration

**Fig. 5:**
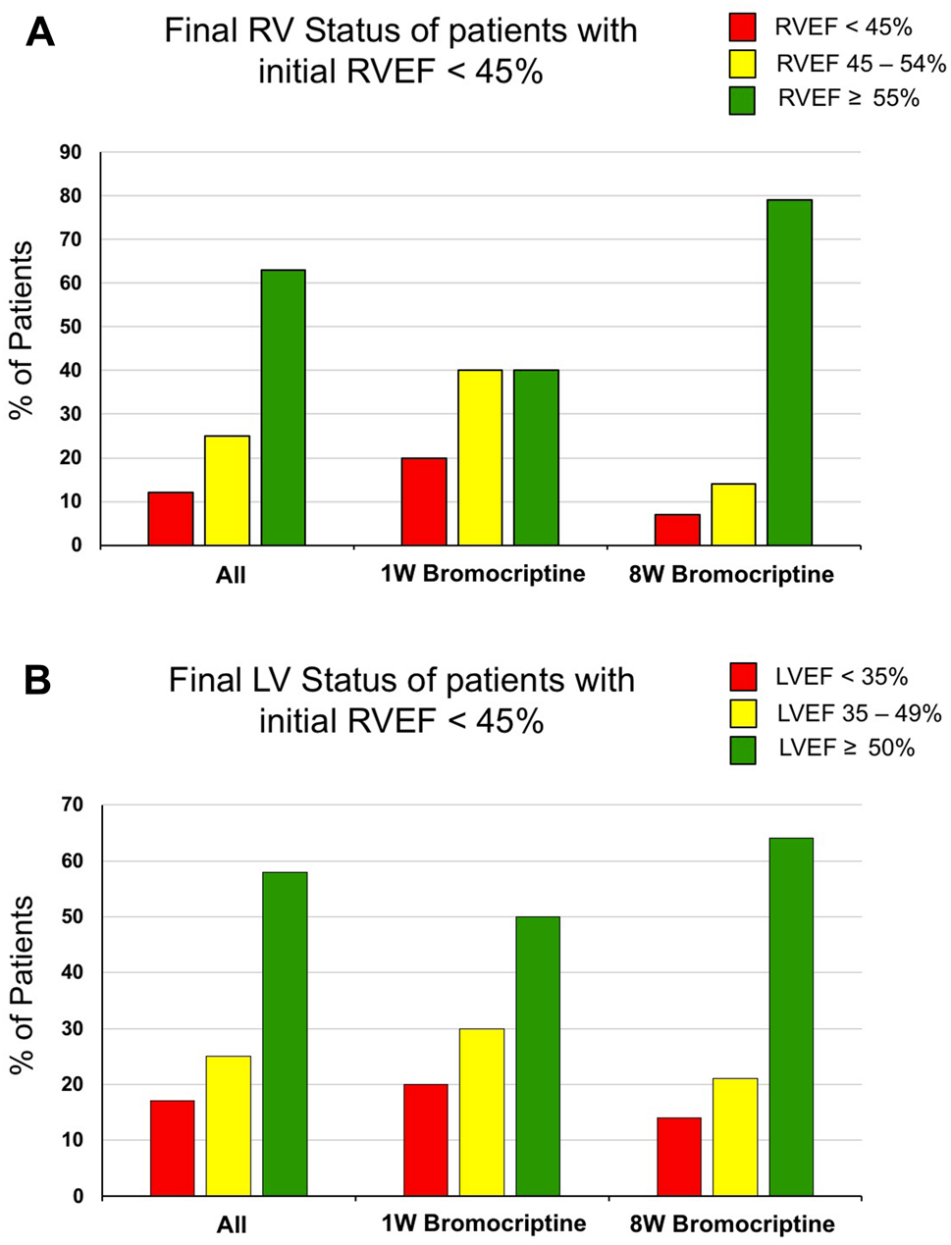
Outcome of patients with initial RV < 45% at 6-month follow-up according to duration of bromocriptine treatment. **a** Right ventricular and **b** left ventricular ejection fraction at 6-month follow-up according to predefined categories in all patients with reduced RVEF at baseline (left bars) and subdivided between patients treated with 1W (center bars) or 8W (right bars) bromocriptine

After 6-month follow-up, 40% (4 of 10) of the patients with RV dysfunction in the 1W group showed full functional RV recovery (RVEF ≥ 55%), 40% (4 of 10) partial recovery (RVEF from 45 to < 54%) and 20% (2 of 10) no recovery (RVEF < 45%). In the 8W arm, the respective rates were 79% (11 of 14) for full functional recovery, 14% (2 of 14) for partial recovery, and 7% (1 of 14) for no recovery (Fig. 5a). Full recovery rates showed a descriptive benefit for the 8W group (79% in 8W group compared with 40% in 1W group), although the difference in the rate of full RV recovery was not statistically significant [two-sided Fisher exact test resulted in *p* = 0.092 with an OR of 0.1818 (95% confidence interval 0.91–33.20)]. As for the left ventricular function, 50% of the patients with RV dysfunction in the 1W group showed full functional LV recovery (LVEF ≥ 50%), 30% partial recovery (LVEF between 35% and < 49%) and 20% no recovery (LVEF < 35%). In the 8W arm, the respective rates were 64% for full functional recovery, 21% for partial recovery, and 15% for no recovery (Fig. 5b). Although 8W bromocriptine treatment achieved a trend for higher rate of full LV recovery after 6 months (64% in 8W group compared with 50% in 1W group), the difference was not statistically significant [two-sided Fisher exact test resulted in *p* = 0.678 with an OR of 0.555 (95% confidence interval 0.1064–2.902), Fig. 5b].

### Correlation between right and left ventricular function at baseline and follow-up in patients with biventricular dysfunction

At baseline, a significant correlation between RVEF and LVEF was found in both the 1W group (*r* = 0.76, *p* = 0.01) and the 8W group (*r* = 0.57, *p* = 0.03) (Suppl. Fig. SI). Interestingly, this correlation was no longer present at follow-up in neither one of the treatment groups (1W: *r* = 0.50, *p* = 0.08; 8W: *r* = 0.32, *p* = 0.27). Moreover, we did not observe a shift of the slopes towards one particular ventricle at follow-up indicating that the treatment did not affect one ventricle more than the other (1W: slope at baseline 0.8; slope at follow-up 0.8; 8W: slope at baseline 0.4, slope at follow-up 0.5).

## Discussion

The present study is the first to test the impact of a specific therapeutic strategy in PPCM patients with RV involvement and provides several important observations: (1) bromocriptine treatment in addition to guideline-based heart failure therapy in PPCM patients with right ventricular involvement is associated with high rate of RV and LV improvement; (2) bromocriptine treatment in this patient population reaches high probability of full RV and LV recovery; and (3) no significant differences were observed between the short-term and long-term bromocriptine treatment regime with regard to RV and LV improvement or full recovery. However, we observed a trend for higher degree of RV and LV improvement as well as higher likelihood of cardiac recovery after 6 months in the long-term bromocriptine treatment group.

Findings from experimental, genetic, clinical and imaging studies over the past two decades have substantially broadened our understanding of both the pathomechanisms as well as the clinical picture of PPCM [2, 13]. In particular, the nursing hormone prolactin and its cleaved 16 kDa form are considered instrumental in the pathophysiology of PPCM [4, 6, 14]. However, despite advances in understanding various aspects of PPCM, so far, clinical trials testing disease-specific therapeutics are limited [1, 2, 15]. Moreover, none of the trials testing therapeutic strategies in PPCM has yet distinguished between different clinical or functional subtypes of PPCM.

We previously found in a prospective registry of a German PPCM cohort using CMR that 35% of the patients had reduced RV systolic function at initial presentation [8]. This estimation was further confirmed by the results of the North American IPAC (Investigations of Pregnancy Associated Cardiomyopathy) study demonstrating that RV function as assessed by echocardiographic fractional area change (FAC) was reduced in 38% of the patients [9]. In our registry, only 25% of the patients with RV dysfunction demonstrated LV recovery at follow-up despite optimal heart failure medication. This observation aligned with the data of the IPAC study in which only 19% of the patients with RV dysfunction revealed LV recovery at follow-up highlighting the clear impact of RV function on the patients’ clinical outcome. The unfavorable outcome of patients with RV dysfunction at baseline was also observed in the current study, although the overall prognosis in this study population was better than reported in previous registries [8, 9]. In view of the results from both registries, the data of the current study suggest a potential therapeutic benefit of bromocriptine in these high-risk patients as 58% of all patients with RV dysfunction reached full cardiac recovery upon bromocriptine treatment in addition to standard heart failure medication regardless of the treatment duration. However, the rather low number of patients and the lack of a placebo arm may be considered as limitation of our study. Furthermore, a placebo group was not permitted by the ethics committee given the results of a previous pilot trial and observational studies [4, 6, 16] and the risk for mastitis if nursing is suddenly stopped without medical support. Thus, the lack of a control group limits the conclusion of a true bromocriptine effect.

It remains to be elucidated whether RV involvement in PPCM constitutes a distinct biventricular subtype with particular pathophysiologic mechanisms affecting the right ventricle or whether RV dysfunction results from impaired LV function. In particular, secondary pulmonary hypertension with subsequent increased RV afterload is a potential mechanism [17]. In many cases PPCM begins in the left ventricle and progresses to involve both right and left sides of the heart, leading to more and more severe biventricular dysfunction. Our findings of reduced stroke volume index argue in favor of RV contractile impairment. This again may be due to PPCM specific microvascular damage of RV myocardium. However, RV dysfunction may also result from decreased RV perfusion based on decreased LV systolic driving pressure, and, thus linked with the severity of LV dysfunction [17]. In this regard, further investigation in both experimental models as well as in clinical studies integrating multiple imaging techniques (e.g. strain analysis and CMR-based perfusion assessment) is needed to further explore detailed underlying mechanisms of RV dysfunction in an effort to optimize individualized therapeutic concepts for these patients.

Notably, in the IPAC cohort a higher percentage of African American patients had reduced RV function compared with Non-African American patients at baseline [9]. Moreover, African American patients failed to demonstrate significant improvement in any RV parameters, whereas women of other ethnic groups had significant improvement in RV functional parameters at follow-up. These findings match with recent reports that African American patients develop a more severe form of PPCM [18] and further support genetic aspects and pathophysiologic differences within subtypes of PPCM with some genetic variants similar to dilated cardiomyopathies [19, 20].

In conclusion, the findings of this study reassure that inhibition of prolactin release with bromocriptine is an effective treatment option also for PPCM patients with RV involvement. The study detected a trend for a benefit of prolonged bromocriptine therapy over short-term therapy in increasing RVEF or LVEF in this patient population. This observed trend for higher likelihood of full cardiac recovery encourages to further test the added value of a more intensive bromocriptine treatment to standard heart failure therapy in a larger outcome trial primarily including PPCM patients with biventricular impairment at initial presentation.

## Electronic supplementary material

Below is the link to the electronic supplementary material.

Supplementary material 1 (DOC 126 KB)

Supplementary material 2 (JPG 137 KB)
