# Supplementary material for: Bromocriptine treatment in patients with peripartum cardiomyopathy and right ventricular dysfunction
Source: Clin Res Cardiol. 2018 Aug 18;108(3):290–7. doi: 10.1007/s00392-018-1355-7 (PMC6394477; doi:10.1007/s00392-018-1355-7)
Supplement: Supplementary file 1 — Supplementary material 1 (DOC 126 KB) [file 392_2018_1355_MOESM1_ESM.doc]

**Supplemental material**

**Table S1. Baseline characteristics of all study patients**

| **Characteristic** | **Patients with RV dysfunction**  **(N=24)** | **Patients without RV dysfunction**  **(N=16)** | **P value** |
| --- | --- | --- | --- |
| Age – yr | 33.0 ± 5.0 | 34.3 ± 4.6 | 0.439 |
| Median Gravida (range) | 3 (1-6) | 1 (1-7) | 0.126 |
| Median Parity (range) | 2 (1-6) | 1 (1-7) | 0.108 |
| Race or ethnic group – no. (%)  Caucasian  Black | 24 (100)  0 (0) | 16 (100)  0 (0) |  |
| Systolic blood pressure – mm Hg | 109 ± 20 | 121 ± 15 | 0.052 |
| Heart rate – beats per minute | 87 ± 11 | 90 ± 15 | 0.523 |
| Body-mass index | 28 ± 6.6 | 29 ± 8.4 | 0.796 |
| Serum creatinine – mg/dl | 0.90 ± 0.1 | 0.76± 0.2 | **0.015** |
| Median NT-proBNP – pg/ml (range) | 2376 (175-21290) | 1956 (367-12503) | 0.477 |
| NYHA functional class – no. (%)  I  II  III  IV | 0 (0)  3 (13)  9 (37)  12 (50) | 0 (0)  3 (19)  3 (19)  10 (62) | 0.667  0.296  0.525 |
| Medical history – no. (%)  Hypertension  Diabetes  Smoker or former smoker | 5 (20)  8 (2)  9 (36) | 4 (25)  0 (0)  9 (56) | 1.000  0.517  0.334 |
| Pregnancy related conditions – no. (%)  Preeclampsia  Gestational diabetes | 2 (8)  2 (8) | 5 (31)  0 (0) | 0.094  0.507 |
| Treatment at randomization – no. (%)  ACE inhibitor  ARB  Mineralcorticoid antagonist  Beta-blocker  Diuretic | 22 (92)  2 (8)  21 (87)  24 (100)  20 (83) | 14 (88)  2 (12)  14 (88)  14 (88)  13 (81) | 1.000  1.000  1.000  0.153  1.000 |

Values are means ± SD. Data were missing for N-terminal pro-B-type natriuretic peptide for 1 patient in the 1W and 1 patient in the 8W bromocriptine group. LVEF: left ventricular ejection fraction; RVEF: right ventricular ejection fraction; NT-proBNP: N-terminal prohormone of brain natriuretic peptide; NYHA: New York Heart Association; ACE: angiotensin-converting enzyme; ARB: angiotensin II receptor blocker.

**Table S2. Baseline characteristics of PPCM patients with RV dysfunction treated with 1 week (1W) vs. 8 weeks (8W) bromocriptine**

| **Characteristic** | **1W Bromocriptine**  **(N = 10)** | **8W Bromocriptine**  **(N = 14)** | **P-value** |
| --- | --- | --- | --- |
| Age – yr | 34.5 ± 5.7 | 33.0 ± 4.3 | 0.359 |
| Median Gravida (range) | 3 (1-7) | 2 (1-5) | 0.528 |
| Median Parity (range) | 2 (1-7) | 2 (1-4) | 0.515 |
| Race or ethnic group – no. (%)  Caucasian  Black | 20 (100)  0 (0) | 24 (100)  0 (0) |  |
| Systolic blood pressure – mm Hg | 119 ± 22 | 109 ± 15 | 0.101 |
| Heart rate – beats per minute | 87 ± 16 | 86 ± 11 | 0.748 |
| Body-mass index | 27.5 ± 6.4 | 29.1 ± 7.8 | 0.493 |
| Serum creatinine – mg/dl | 0.8 ± 0.2 | 0.9 ± 0.2 | **<0.001** |
| Clinical features of heart failure  LVEF – %  RVEF – %  Median NT-proBNP – pg/ml (range) | 24 ± 9  36 ± 9  1974 (175-13463) | 24 ± 6  34 ± 10  2492 (367-21290) | 0.739  0.204  0.297 |
| NYHA functional class – no. (%)  I  II  III  IV | 0 (0)  3 (15)  4 (20)  13 (65) | 0 (0)  4 (17)  8 (33)  12 (50) | 1.000  0.498  0.371 |
| Medical history – no. (%)  Hypertension  Diabetes  Smoker or former smoker | 3 (15)  2 (10)  9 (45) | 6 (25)  0 (0)  15 (63) | 0.477  0.201  0.362 |
| Pregnancy related conditions – no. (%)  Preeclampsia  Gestational diabetes | 3 (15)  3 (15) | 5 (21)  0 (0) | 0.710  0.086 |
| Treatment at randomization – no. (%)  ACE inhibitor  ARB  Mineralcorticoid antagonist  Beta-blocker  Diuretic | 19 (95)  1 (5)  17 (85)  17 (85)  17 (85) | 20 (83)  3 (13)  20 (79)  24 (100)  21 (88) | 0.356  0.614  1.000  0.086  1.000 |

Plus–minus values are means ± SD. Data were missing for N-terminal pro-B-type natriuretic peptide for 1 patient in the 1W and 1 patient in the 8W bromocriptine group. LVEF: left ventricular ejection fraction; RVEF: right ventricular ejection fraction; NT-proBNP: N-terminal prohormone of brain natriuretic peptide; NYHA: New York Heart Association; ACE: angiotensin-converting enzyme; ARB: angiotensin II receptor blocker.

**Table S3. Baseline CMR parameters in PPCM with or without RV dysfunction**

| **Parameters:** | **Patients with RV dysfunction**  **(N=24)** | **Patients without RV dysfunction**  **(N=16)** | **P value** |
| --- | --- | --- | --- |
| **LV Parameters** |  |  |  |
| LVEF (%) | 25 ± 8 | 36 ± 7 | **<0.001** |
| LV-EDV/BSA (ml/m^2^) | 140 ± 27 | 121 ± 34 | 0.098 |
| LV-ESV/BSA (ml/m^2^) | 107 ± 27 | 67 ± 18 | **<0.001** |
| LV-SV/BSA (ml/m^2^) | 36 ± 18 | 41 ± 10 | 0.312 |
| LV-Mass/BSA (g/m^2^) | 90 ± 15 | 76 ± 17 | **0.010** |
| **RV Parameters** |  |  |  |
| RVEF (%) | 35 ± 9 | 57 ± 6 | **<0.001** |
| RV-EDV/BSA (ml/m^2^) | 96 ± 22 | 73 ± 14 | **0.001** |
| RV-ESV/BSA (ml/m^2^) | 60 ± 20 | 32 ± 10 | **<0.001** |
| RV-SV/BSA (ml/m^2^) | 34 ± 9 | 41 ± 7 | **0.014** |
| RV-Mass/BSA (g/m^2^) | 29 ± 11 | 25 ± 7 | **0.018** |

Right ventricular ejection fraction (RVEF), left ventricular ejection fraction (LVEF), end-diastolic volume/BSA (EDV/BSA), end-systolic volume/BSA (ESV/BSA), stroke volume to body surface area (SV/BSA), mass/BSA (Mass/BSA).

**Table S4. Baseline CMR parameters in PPCM with RV dysfunction** treated with 1 week (1W) vs. 8 weeks (8W) bromocriptine

| **Parameters:** | **1W Bromocriptine**  **(N = 10)** | **8W Bromocriptine**  **(N = 14)** | **P-value** |
| --- | --- | --- | --- |
| **LV Parameters** |  |  |  |
| LVEF (%) | 24 ± 9 | 24 ± 6 | 0.851 |
| LV-EDV/BSA (ml/m^2^) | 147 ± 24 | 134 ± 25 | 0.359 |
| LV-ESV/BSA (ml/m^2^) | 111 ± 24 | 105 ± 25 | 0.703 |
| LV-SV/BSA (ml/m^2^) | 43 ± 26 | 31 ± 6 | 0.116 |
| LV-Mass/BSA (g/m^2^) | 95 ± 19 | 91 ± 9 | 0.605 |
| **RV Parameters** |  |  |  |
| RVEF (%) | 36 ± 9 | 34 ± 10 | 0.509 |
| RV-EDV/BSA (ml/m^2^) | 97 ± 27 | 94 ± 19 | 0.719 |
| RV-ESV/BSA (ml/m^2^) | 59 ± 18 | 62 ± 22 | 0.565 |
| RV-SV/BSA (ml/m^2^) | 37 ± 10 | 33 ± 9 | 0.290 |
| RV-Mass/BSA (g/m^2^) | 35 ± 12 | 33 ± 7 | 0.565 |

Right ventricular ejection fraction (RVEF), left ventricular ejection fraction (LVEF), end-diastolic volume/BSA (EDV/BSA), end-systolic volume/BSA (ESV/BSA), stroke volume to body surface area (SV/BSA), mass/BSA (Mass/BSA).

**Table S5: Tests on between-groups effects of RVEF in all patients at 6 months follow-up**

| Source | Square sum of type III | df | Mean of square | F | Sig. | Partial eta-squared | Decentrality parameter | Observed  power |
| --- | --- | --- | --- | --- | --- | --- | --- | --- |
| Corrected model | 596,704 | 2 | 298,352 | 2,518 | 0,092 | 0,101 | 5,035 | 0,479 |
| Intercept | 5149,546 | 1 | 5149,546 | 43,455 | 0,000 | 0,491 | 43,455 | 1 |
| RVEF_BLr | 588,87 | 1 | 588,87 | 4,969 | 0,031 | 0,099 | 4,969 | 0,588 |
| **Treatment arm** | **36,452** | **1** | **36,452** | **0,308** | **0,582** | **0,007** | **0,308** | **0,084** |
| Error | 5332,608 | 45 | 118,502 |  |  |  |  |  |
| Total | 125031 | 48 |  |  |  |  |  |  |
| Total corrected variation | 5929,313 | 47 |  |  |  |  |  |  |

**Table S6: Tests on between-groups effects of LVEF in patients with biventricular involvement at 6 months follow-up**

| Source | Square sum of type III | df | Mean of square | F | Sig. | Partial eta-squared | Decentrality parameter | Observed  power |
| --- | --- | --- | --- | --- | --- | --- | --- | --- |
| Corrected model | 1385,023 | 2 | 692,512 | 6,027 | 0,007 | 0,309 | 12,054 | 0,844 |
| Intercept | 1909,685 | 1 | 1909,685 | 16,620 | 0,000 | 0,381 | 16,620 | 0,975 |
| LVEF_BLr | 1316,573 | 1 | 1316,573 | 11,458 | 0,002 | 0,298 | 11,458 | 0,904 |
| **Treatment arm** | **188,529** | **1** | **188,529** | **1,641** | **0,211** | **0,057** | **1,641** | **0,235** |
| Error | 3102,344 | 27 | 114,902 |  |  |  |  |  |
| Total | 72937,000 | 30 |  |  |  |  |  |  |
| Total corrected variation | 4487,367 | 29 |  |  |  |  |  |  |

**Table S7: Tests on between-groups effects of RVEF in patients with biventricular involvement at 6 months follow-up**

| Source | Square sum of type III | df | Mean of square | F | Sig. | Partial eta-squared | Decentrality parameter | Observed  power |
| --- | --- | --- | --- | --- | --- | --- | --- | --- |
| Corrected model | 317,491 | 2 | 158,745 | 1,953 | 0,167 | 0,157 | 3,907 | 0,358 |
| Intercept | 2988,767 | 1 | 2988,767 | 36,779 | 0,000 | 0,637 | 36,779 | 1,000 |
| LVEF_BLr | 130,805 | 1 | 130,805 | 1,610 | 0,218 | 0,071 | 1,610 | 0,228 |
| **Treatment arm** | **216,038** | **1** | **216,038** | **2,659** | **0,118** | **0,112** | **2,659** | **0,343** |
| Error | 1706,509 | 21 | 81,262 |  |  |  |  |  |
| Total | 77288,000 | 24 |  |  |  |  |  |  |
| Total corrected variation | 2024,000 | 23 |  |  |  |  |  |  |
