# Supplementary figures and images for: Bromocriptine treatment in patients with peripartum cardiomyopathy and right ventricular dysfunction
Source: Clin Res Cardiol. 2018 Aug 18;108(3):290–7. doi: 10.1007/s00392-018-1355-7 (PMC6394477; doi:10.1007/s00392-018-1355-7)

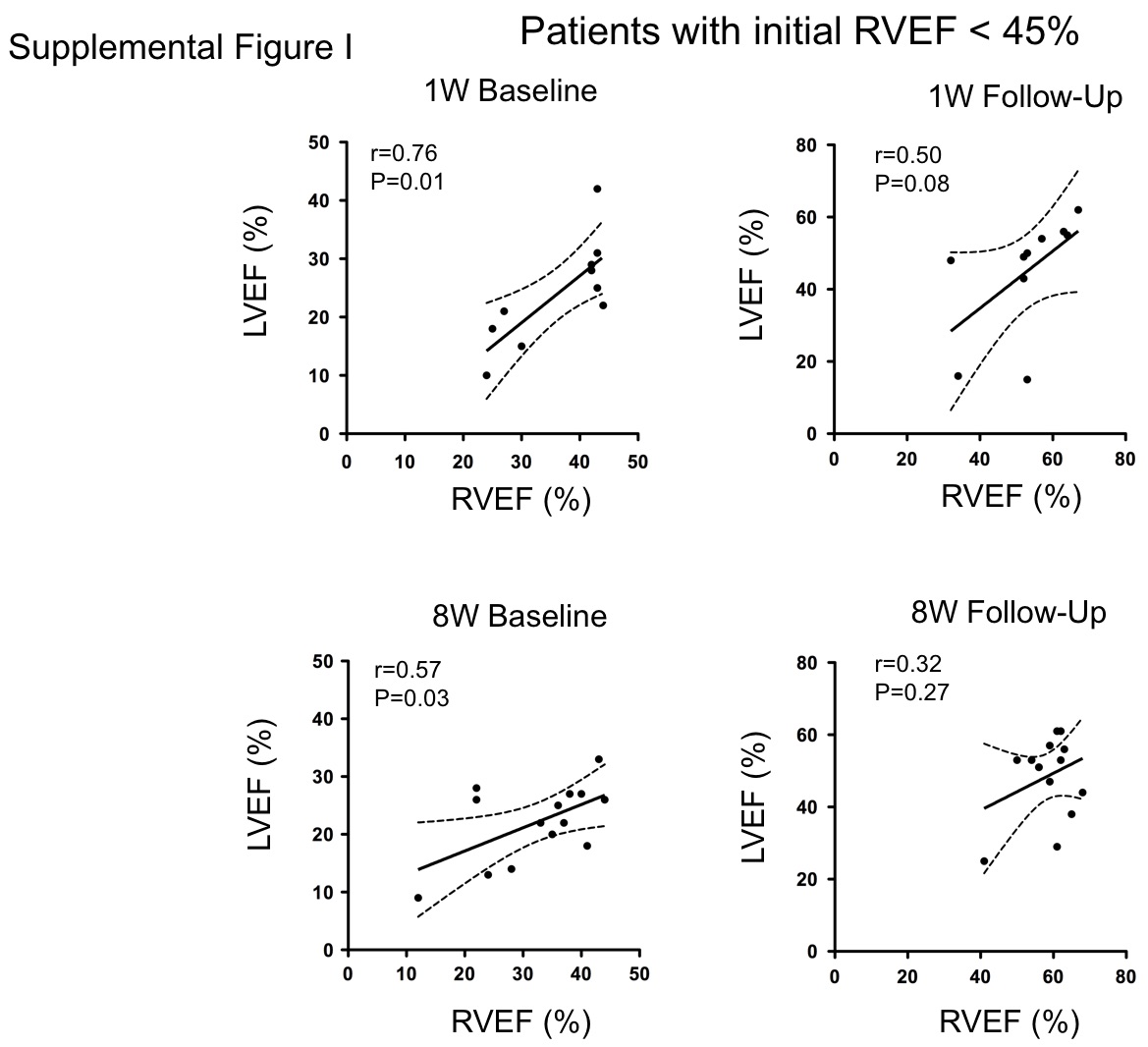

Supplement: Supplementary file 2 — Supplementary material 2 (JPG 137 KB) [file 392_2018_1355_MOESM2_ESM.jpg]
